# First-degree Relatives of Celiac Disease Patients Have Increased Seroreactivity to Serum Microbial Markers

**DOI:** 10.3390/nu12041073

**Published:** 2020-04-13

**Authors:** Liisa Viitasalo, Sari Iltanen, Heini Huhtala, Päivi Saavalainen, Katri Kaukinen, Katri Lindfors, Kalle Kurppa

**Affiliations:** 1 Centre for Child Health Research and Department of Pediatrics, Tampere University Hospital, FI-33521 Tampere, Finland; liisa.viitasalo@tuni.fi (L.V.); sari.iltanen@lshp.fi (S.I.); 2 Laboratory of Genetics, HUS Diagnostic Center, Helsinki University Hospital, FI-00029 Helsinki, Finland; 3 Lapland Central Hospital, FI-96101 Rovaniemi, Finland; 4 Faculty of Social Sciences, Tampere University, FI-33520 Tampere, Finland; heini.huhtala@tuni.fi; 5 Research Program Unit, Immunobiology, and Department of Medical and Clinical Genetics, University of Helsinki, FI-00014 Helsinki, Finland; paivi.saavalainen@helsinki.fi; 6 Celiac Disease Research Centre, Faculty of Medicine and Health Technology, Tampere University, FI-33520 Tampere, Finland; katri.kaukinen@tuni.fi (K.K.); katri.lindfors@tuni.fi (K.L.); 7 Department of Internal Medicine, Tampere University Hospital, FI-33521 Tampere, Finland; 8 Department of Pediatrics, Seinäjoki University Hospital and the University Consortium of Seinäjoki, FI-60320 Seinäjoki, Finland

**Keywords:** celiac disease, relatives, microbiota, *Saccharomyces cerevisiae*, *Pseudomonas fluorescens*, *Bacteroides caccae*

## Abstract

Risk of celiac disease (CD) is increased in relatives of CD patients due to genetic and possible environmental factors. We recently reported increased seropositivity to anti-*Saccharomyces cerevisiae* (ASCA), *Pseudomonas fluorescens*-associated sequence (anti-I2) and *Bacteroides caccae* TonB-linked outer membrane protein (anti-OmpW) antibodies in CD. We hypothesized these markers also to be overrepresented in relatives. Seropositivity and levels of ASCA, anti-I2 and anti-OmpW were compared between 463 first-degree relatives, 58 untreated and 55 treated CD patients, and 80 controls. CD-associated human leukocyte antigen (HLA)-haplotypes and transglutaminase (tTGab) and endomysium (EmA) antibodies were determined. One or more of the microbial antibodies was present in 75% of relatives, 97% of untreated and 87% of treated CD patients and 44% of the controls. The relatives had higher median ASCA IgA (9.13 vs. 4.50 U/mL, *p* < 0.001), ASCA IgG (8.91 vs. 5.75 U/mL, *p* < 0.001) and anti-I2 (absorbance 0.74 vs. 0.32, *p* < 0.001) levels than controls. There was a weak, positive correlation between tTGab and ASCA (r = 0.31, *p* < 0.001). Seropositivity was not significantly associated with HLA. To conclude, seropositivity to microbial markers was more common and ASCA and anti-I2 levels higher in relatives of CD patients than controls. These findings were not associated with HLA, suggesting the role of other genetic and environmental factors.

## 1. Introduction

Celiac disease (CD) is an immune-mediated condition characterized by gluten-induced small-bowel enteropathy. Almost all patients carry human leukocyte antigen (HLA) alleles encoding DQ2 or DQ8 heterodimers [1]. These alleles are nevertheless also present in up to 35% of the general population and do not fully explain the genetic risk [2]. Recent genome-wide association studies and immunogenetic studies have identified numerous non-HLA loci and single nucleotide polymorphisms that may modify CD risk [3,4]. Partly due to shared genetic predisposition, the relatives of patients have an increased susceptibility to CD, the average prevalence among first-degree relatives being approximately 8% [5] compared with 1%–2% in the general population [6,7].

However, only a minority of at-risk individuals develop CD, and the concordance even varies between identical twins [8,9], which implicates environmental factors. The prevalence may also vary between adjacent countries with similar genetic backgrounds and gluten consumption [10], and retrospective measurements of stored samples indicate a rise in the true incidence [6,11,12]. As one potentially associated factor, the role of intestinal microbiota in the development of CD has aroused particular interest [13,14,15]. Previously, we and others observed elevated levels of antibodies to microbial markers *Saccharomyces cerevisiae* (ASCA), *Pseudomonas fluorescens*-associated sequence (anti-I2) and *Bacteroides caccae* TonB-linked outer membrane protein (anti-OmpW) in inflammatory bowel disease [16,17,18]. We have shown increased seroreactivity to these markers also in overt CD [19] and a decrease of the antibody levels during gluten-free diet (GFD) [20]. Further, these microbial markers are detectable in early stages of the disease even before the presence of villous atrophy and serum CD-specific autoantibodies [21].

We hypothesized that close relatives of CD patients, with partially shared living environments and genetic factors, could have increased seroreactivity to microbial markers. This was investigated by comparing their frequency of seropositivity and levels of microbial antibodies with those in untreated and treated CD patients and in healthy controls.

## 2. Materials and Methods 

### 2.1. Study Participants

The study was carried out at Tampere University and Tampere University Hospital. Previously diagnosed CD patients were recruited in a nationwide search through newspaper advertisements and via patient societies. Their medical records were obtained with permission, and only subjects with a biopsy-proven diagnosis were included. Relatives of these patients were invited to a screening study comprising personal interviews and measurement of CD serology. Additional blood samples were drawn for research purposes. Exclusion criteria for the relatives were previously diagnosed CD or dermatitis herpetiformis, or otherwise initiated gluten-free diet (GFD). Altogether, 3031 relatives met the inclusion criteria and entered the original screening study. Duodenal biopsy was offered for all relatives with positive CD serology. For the present study, serum samples from 463 first-degree relatives were randomly selected for the measurement of ASCA, anti-I2 and anti-OmpW. The CD control group comprised 58 biopsy-proven patients who underwent measurements of the CD serology and microbial markers at diagnosis and after one year on GFD (*n* = 55). In addition, 80 adult blood donors with negative CD serology served as non-CD controls.

### 2.2. CD Autoantibodies and Genotyping

Serum immunoglobulin A (IgA) class endomysium autoantibodies (EmA) were tested by an indirect immunofluorescence method using human umbilical cord as substrate [22]. Titers 1: ≥ 5 were deemed positive and diluted up to 1:4000 or until negative. Serum IgA class tissue transglutaminase autoantibodies (tTGab) were measured by an enzyme-linked immunosorbent assay (ELISA, INOVA diagnostics, San Diego, CA) according to the manufacturer’s instructions. A cutoff ≥ 30 U/mL was applied for seropositivity. Some of the CD autoantibody-positive relatives declined the biopsy, but, due to the high specificity of EmA/tTGab [23], the vast majority of them are also likely to have CD. They were therefore analyzed as a separate group.

The CD-associated HLA DQ haplotypes (DQ2.5, DQ2.2, DQ8) were determined from the relatives and CD patients with the tagging single nucleotide polymorphism method or with the Olerup SSP DQ low-resolution kit (Olerup SSP AB, Stockholm, Sweden) as described elsewhere [24,25].

### 2.3. Microbial Antibodies

Serum IgA and IgG class ASCA were measured by a commercial ELISA (Quanta Lite ASCA, INOVA Diagnostics Inc., San Diego, CA) considering levels ≥ 25 U/mL positive. *E. coli* XL-1 blue and *E. coli* BL-21 (Stratagene, La Jolla, CA) strains and previously reported antigen purification techniques [26,27] were used to produce I2-GST and OmpW antigens. The serum samples were diluted 1:50, and IgA anti-I2 and anti-OmpW antibodies were measured with an in-house ELISA. For anti-I2, the cutoff level for positivity was set at absorbance 0.5. For anti-OmpW, it was set at 0.6 in children and 1.0 in adults based on our previous studies showing age differences in the normal range [16,19].

### 2.4. Statistical Analysis

Quantitative data are shown in tables as percentages or as medians with lower and upper quartiles. The data were cross-tabulated in order to ascertain the overlap of seropositivity for microbial antibodies in different study groups. The Kruskal–Wallis test was used to compare the differences in microbial antibody levels between the groups. Correlations between autoantibodies and microbial markers were tested with Spearman’s rank correlation coefficient. Associations in the seropositivity to microbial antibodies within and between the families were also tested. The chi-square statistic for the change in the -2 log-likelihood from the constant only model to the model with “family” was used to determine whether the inclusion of “family” contributed significantly to model fit. A *p* value < 0.05 was considered significant. Statistical analyses were carried out with SPSS Statistics for Windows (IBM Corp., Armonk, NY, USA).

### 2.5. Ethical Aspects

The study protocol was approved by the Ethics Committee of the Pirkanmaa Hospital District, study identification code ETL R05183. All participants or, in the case of children, their legal guardians gave written informed consent. The paper follows the rules of the Declaration of Helsinki.

## 3. Results

The gender distribution was fairly equal among the relatives, whereas a majority of CD patients were women, and there were more men in the non-CD control group (Table 1). There were no major differences in the median ages between the groups (Table 1), but 49 (10.6%) of the relatives were <18 years of age, while the other groups comprised only adults.

**Figure 1:**
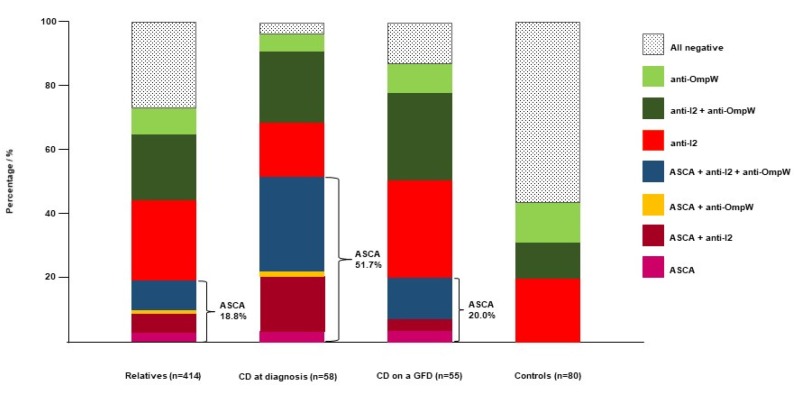
Distribution of seropositivity to antibodies against *Saccharomyces cerevisiae* (ASCA), *Pseudomonas fluorescens*-associated sequence (anti-I2 antibodies) and *Bacteroides caccae* TonB-linked outer membrane protein (anti-OmpW antibodies) among autoantibody-negative relatives of celiac disease (CD) patients, CD patients (at diagnosis and on a GFD) and controls.

The relatives were divided into CD autoantibody-negative (*n* = 414) and autoantibody-positive (*n* = 49) groups and were analyzed separately (Table 1). Among the autoantibody-negative relatives, seropositivity for at least one of the microbial markers was more common than in the non-CD controls but less frequent than in the CD patients (Figure 1). The most notable difference was seen in ASCA, as 19% of the relatives without CD-autoantibodies and none of the controls were seropositive for ASCA IgA, ASCA IgG, or both. In addition, anti-I2 and anti-OmpW positivity was more common among the autoantibody-negative relatives than controls (61% and 40% vs. 31% and 24%, respectively; Figure 1).

**Figure 2:**
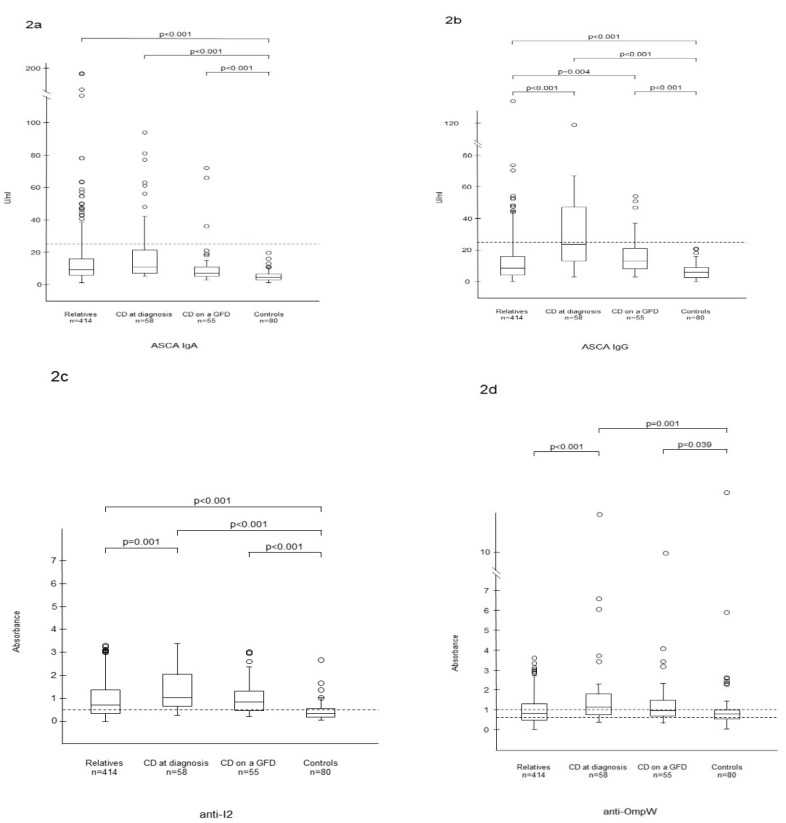
Serum levels of antibodies to *Saccharomyces cerevisiae* (ASCA) in IgA (**a**) and IgG (**b**) classes, *Pseudomonas fluorescens*-associated sequence (anti-I2) (**c**) and *Bacteroides caccae* TonB-linked outer membrane protein (anti-OmpW) (**d**) in autoantibody-negative relatives. Horizontal lines indicate the cutoff level for seropositivity of each antibody.

The median levels of ASCA IgA, ASCA IgG and anti-I2 were also significantly higher in the autoantibody-negative relatives than those in the control group (Figure 2a–c), whereas anti-OmpW was higher only in untreated and treated CD patients (Figure 2d). ASCA IgG was higher in both untreated and treated CD patients and anti-I2/OmpW in untreated patients when compared with autoantibody-negative relatives (Figure 2b–d). 

Altogether, 46 out of the 49 autoantibody-positive relatives had HLA-DQ2 haplotype, DQ8 haplotype, or both. As many as 86% of them showed seroreactivity to at least one microbial marker compared to 73% of the CD antibody-negative relatives, and the median levels of the microbial antibodies were also higher (ASCA IgA 11.1 vs. 8.90 U/mL, *p* = 0.019; ASCA IgG 12.8 vs. 8.37 U/mL, *p* = 0.001; absorbance for anti-I2 0.93 vs. 0.71, *p* = 0.320 and for anti-OmpW 1.00 vs. 0.81, *p* = 0.022, respectively). In contrast to the autoantibody-negative group, anti-OmpW levels were also significantly higher than in the controls (absorbance 0.79, *p* = 0.043).

Adjusting for age and gender or exclusion of children from the comparisons did not affect the results of the prevalence of seropositivity nor median levels of the microbial markers, although the medians were significantly lower in children than in adults (ASCA IgA 6.30 vs. 9.64 U/mL, *p* < 0.001; ASCA IgG 7.13 vs. 9.18 U/mL, *p* = 0.070; absorbance for anti-I2 0.34 vs. 0.79, *p* < 0.001 and for anti-OmpW 0.54 vs. 0.87, *p* < 0.001, respectively).

Seropositivity to anti-I2 and anti-OmpW was significantly more frequent between relatives in the same family than between different families (*p* < 0.001 for anti-I2 and *p* = 0.001 for anti-OmpW, respectively). In ASCA, this was observed only when autoantibody-positive relatives were also included in the analysis (*p* = 0.007).

There were no significant differences in the distribution of seropositivity across microbial markers when the relatives were categorized according to their HLA haplotypes (Table 2).

There was a weak, positive correlation between the values of tTGab and ASCA IgA (r = 0.31, *p* < 0.001), whereas correlation coefficients between the other microbial markers and tTGab or EmA were <0.3.

## 4. Discussion

The main finding of the present study was increased seroreactivity to microbial markers in the relatives of CD patients compared with controls even after the exclusion of CD autoantibody-positive individuals. This was observed particularly with ASCA and anti-I2, the median levels of which were also significantly higher than levels in the controls, although they were lower than in CD patients. To the best of our knowledge, the only study to report on this issue so far was a conducted by Da Silva et al., who investigated seropositivity to ASCA in relatives of CD patients [28]. They divided 76 relatives into EmA/tTGab negative and positive groups, while 57 individuals with negative CD autoantibodies and no family risk served as controls. Partly in contrast to us, there was a significantly higher frequency of positivity to ASCA IgA/G only in autoantibody-positive relatives compared with the controls [28]. This discrepancy may, at least in part, be explained by the smaller number of participants since there was a trend toward overrepresentation of ASCA, also among the CD autoantibody-negative relatives. There may also have been methodological differences, as the authors did not report the kits used for the ASCA measurements.

Owing to the high specificity of tTGab and EmA [23], most of the autoantibody-positive relatives were likely CD patients. Therefore, their increased seroreactivity to microbial markers is logically in line with that observed in already-diagnosed CD. By contrast, the increased frequency of seroreactivity to a part of the microbial markers in the autoantibody-negative relatives is not as easily explained. It is to be noted that Setty and colleagues [29] previously reported that tTGab-negative relatives of CD patients had signs of intestinal epithelial stress, demonstrated by ultrastructural alterations of microvilli, and increased expression of heat shock proteins and interleukin-15 along with elevated expression of activating NK receptors on intraepithelial cytotoxic T cells. Thus, even in the absence of CD autoantibodies or characteristic histological damage to the intestine, at least some of the relatives appeared to display proinflammatory responses reminiscent of CD. This raises the question of whether the observed abnormal microbial antibody production could also be implicated in this process.

Setty et al. also speculated about a possible genetic predisposition to epithelial stress [29] and suggested a possible HLA and other as yet-unidentified genetic associations. We observed no significant association between the distribution of ASCA, anti-I2 and anti-OmpW positivity and the CD-related HLA haplotypes, suggesting that at least HLA genetics does not markedly affect the serological response. In line with this, HLA DQ2/8 are not overexpressed in inflammatory bowel disease (IBD) patients [30] who also may have increased seropositivity to microbial markers [16,17]. Genetics may still play a role in microbial antibody production in intestinal diseases, as demonstrated by two studies comparing levels of microbial antibodies between monozygous and dizygous twin pairs with IBD. Amcoff et al. reported that the differences in the anti-I2 antibody levels were smaller within than between monozygous twin pairs, even if only one of them had IBD [31]. However, this was not seen in dizygous twins with one suffering from IBD and the other being healthy and having partly discordant genetics, supporting the role of genetic factors [31]. By contrast, similar ASCA levels were observed only in a subgroup of monozygous twins both having IBD [31,32]. Bearing this in mind, it is interesting that we found stronger associations of anti-I2 positivity between the relatives from the same family than between the families, whereas with ASCA this was seen only when autoantibody-positive relatives were included in the analysis. Taken together, it seems that both genetic and environmental factors have a role in the antibody production, with this varying depending on the microbial marker, but further studies are needed.

Environmental factors including gluten intake [33,34] and infections in early life [35,36,37] have also been associated with increased CD risk. Other suggested, although controversial [38,39], risk factors include bacterial infections and frequent use of antibiotics [40,41]. Interestingly, the incidence has been reported to vary depending on socioeconomic circumstances [10], leading to the hypothesis that slight microbial exposure increases CD risk by driving immune reactions toward autoantigens and dietary components [42]. Close relatives usually share the living milieu and may, thus, experience similar environmental modulatory effects on the microbiota and immune system that, in addition to genetics, could give rise to parallel responses to microbial antigens. It remains unclear, however, which external factors drive these responses and whether the microbial markers have a causal role [43]. It is likely that a complex interaction between multiple factors, such as dysregulation of the immune system, changes in the epithelial barrier, and dysbiosis causes the loss of tolerance to microbial antigens [13,44,45,46]. In addition, a very recent study showed that *Pseudomonas fluorescens* peptides mimic gluten epitopes and activate gliadin-reactive T cells, with this cross-reactivity possibly contributing to the onset of CD [47].

We previously found most of the potential CD patients to already exhibit the microbial markers before the development of villous damage or autoantibodies [21], reflecting the situation in the relatives in the present study. Interestingly, Torres and colleagues recently showed that ASCA also predicts forthcoming Crohn’s disease up to five years before the diagnosis [48]. More studies are needed to determine the role of these markers in early development of CD and whether they could be utilized to predict the disease in at-risk groups. 

The main strengths of our study include the large and well-defined cohort of relatives of CD patients who underwent systematic screening for CD-associated HLA and autoantibodies and the representative control groups. As a weakness, however, large differences between the group sizes could have influenced the results. Furthermore, only the groups with relatives contained pediatric subjects, although the results remained unchanged after excluding children from the analyses. Genetic data of the non-HLA alleles were also lacking, which could be an even more significant limitation among relatives with a less marked HLA predisposition to CD. Since we did not have detailed information on the health condition of the relatives, and the histological status of their intestines remains unknown, it is possible that some of them had unreported CD or another disease affecting the results. Furthermore, dietary data of the relatives was lacking, and it is possible that cross-reactions between food antigens influenced the microbial antibody levels. ASCA is known to cross-react with other yeast strains [49], and the lack of correlation between ASCA antibodies and *Saccharomyces cerevisiae* DNA on intestinal mucosa [50] indicates the possibility of some yet-unidentified cross-reactive antigens. In accord with our previous study [51], for currently unclear reasons, ASCA levels were generally higher in the IgG class than the IgA class. By contrast, IgA class ASCA seems to be more consistently elevated in IBD [48,52]. Which of these two antibody classes is the more useful marker in CD would be an interesting subject for further research. The median duration of GFD in the CD group was only one year, which may have biased the serological results, as histological and serological recovery often take longer despite a strict diet [53]. Finally, a few adults here had surprisingly high anti-OmpW values compared with our previous studies. Although we still believe that the used cutoff was valid, we recommend that it be confirmed in other populations. 

In conclusion, we found increased seroreactivity to serum microbial markers, particularly ASCA and anti-I2, in relatives of CD patients even in the absence of the disease-specific autoantibodies or other signs of active CD. This observation was not explained by the presence or absence of predisposing HLA haplotypes, thereby suggesting the role of other genetic and environmental factors.

## Figures and Tables

**Table 1:** Demographic data on relatives of celiac disease (CD) patients, CD patients and non-celiac controls.

	Seropositive Relatives	Seronegative Relatives *	CD at Diagnosis	CD on GFD	Non-CD Controls
	*n* = 49	*n* = 414	*n* = 58	*n* = 55	*n* = 80
Females, %	42.9	57.2	77.6	76.4	35.0
Age, median (quartiles), y	41 (31–54)	42 (28–59)	45 (36–59)	46 (38-60)	41 (31–56)

* Negative serum endomysium (titer 1: < 5) and tissue transglutaminase (< 30 U/mL) antibodies. GFD, gluten-free diet.

**Table 2:** Frequency of seropositivity to microbial markers in autoantibody-negative relatives of celiac disease patients with different human leukocyte antigen (HLA) haplotypes.

	DQ2 *n* = 233	DQ8 *n* = 67	DQ2 + DQ8 *n* = 8	DQ2/8 Negative *n* = 89
	%	%	%	%
ASCA IgA	11.2	10.4	12.5	10.1
ASCA IgG	12.9	13.4	0	14.6
Anti-I2	58.4	61.2	75.0	66.3
Anti-OmpW	39.5	35.8	25.0	43.8

ASCA, Anti-*Saccharomyces cerevisiae* antibodies; anti-I2, antibodies to *Pseudomonas fluorescens*-associated sequence; anti-OmpW, antibodies to *Bacteroides Caccae* TonB-linked outer membrane protein; DQ2, HLA-DQA1*05-DQB1*02 (DQ2.5) or HLA-DQA1*02-DQB1*02 (DQ2.2); DQ8, HLA-DQA1*03-DQB1*0302. There were no statistically significant differences between the groups in the distribution of seropositivity.
